# Chronic active EBV infection mimicking periodic fever syndromes: a new challenge for the paediatrician

**DOI:** 10.1186/1546-0096-12-S1-P168

**Published:** 2014-09-17

**Authors:** Roberta Caorsi, Antonella Buoncompagni, Francesca Minoia, Paolo Picco, Sara Signa, Silvia Federici, Martina Finetti, Alberto Martini, Marco Gattorno

**Affiliations:** 1 2nd Division of Pediatrics, Istituto Gaslini, Genoa, Genova, Italy

## Introduction

Chronic active EBV (CAEBV) infection is a rare condition associated to a chronic activation of Epstein Barr virus and therefore to a chronic and potentially lifethreating lymphoproliferation. Few cases of this condition have been described in the Asian population (with a prevalence of expansion of T and NK lymphocytes) and in North America (with a prevalence of expansion of B cells). This condition presents a poor prognosis: most of the patients not treated with bone marrow transplantation die for lymphatic malignancies.

## Objectives

To describe the clinical course of two patients with CAEB, initially misdiagnosed and treated as periodic fever syndromes.

## Methods

We describe the clinical course of two patients presenting with a condition of periodic fever due to the chronic activation of EBV virus.

## Results

A boy of consanguineous parents at the age of 15 months presented, in complete wellbeing, a febrile episode associated to tonsillitis with exudates, lateral cervical lymphadenopathy and hepatosplenomegaly; the blood test revealed an infectious mononucleosis. The clinical course of the disease was regular. In the following months the child presented recurrent episodes of high fever with exudative tonsillitis, adenitis, splenomegaly and sweating, lasting 3-5 days and treated with NSAIDS or antibiotics. The blood examinations revealed neurofilic leukocytosis and elevation of acute phase reactants. An autoinflammatory condition with periodic fever was suspected and therefore on-demand steroidal treatment was suggested; this therapeutic approach was only partially effective. In the following months the boy continued to present periodic fever, occasionally associated to respiratory infections requiring antibiotics, and recurrent episodes of cheratitis. Several destructive dental caries were found as well as hyper sensibility to mosquitoes bite. In light of the persistence of the symptoms, monogenic periodic fevers were ruled out and immunologic test were performed: the level of plasmatic immunoglobulins was reduced and the lymphocytic count revealed an increase of CD20+ cells; the EBV PCR revealed 25000 copies for 100000 leucocytes with prevalence of infection in the B cells.

A second non-related patient of non-consanguineous parents, at the age of four years started to present recurrent episodes of high-grade fever with pharyngitis, oral aftosis and abdominal pain with normal or slightly increased inflammatory markers. The genetic test for periodic fever revealed the presence of the R202Q variant in homozygosis in the *MEFV* gene; the diagnosis of FMF was point out and treatment with colchicine was started, without a clear improvement of the clinical picture. Due to an increase of the transaminase and reduction of the platlets’ count, a bone marrow aspiration was performed, negative for malignancies. Several destructive dental caries were found requiring the avulsion of 11 teeth. Immunologic tests were then performed: immunoglobulis were normal, while the lynphocyte populations’s count revealed a relevant increase of the NK cells, with reduction of the other populations. Virologic investigations revealed a high replication of EBV virus (600000 copies for 100000 monocytes) with a prevalence of infection of NK cells.

## Conclusion

Chronic active EBV infection is a rare condition, rarely individuated in the childhood. However this disease has to be ruled out in paediatric patients with periodic fever syndromes, especially in case of presence of unusual symptoms.

## Disclosure of interest

None declared.

